# Disrupted default mode network connectivity in bipolar disorder: a resting-state fMRI study

**DOI:** 10.1186/s12888-024-05869-y

**Published:** 2024-06-07

**Authors:** Lei Zhao, Qijing Bo, Zhifang Zhang, Feng Li, Yuan Zhou, Chuanyue Wang

**Affiliations:** 1 The National Clinical Research Center for Mental Disorders & Beijing Key Laboratory of Mental Disorders, Beijing Institute for Brain Disorders Center of Schizophrenia, Beijing Anding Hospital, Capital Medical University, No.5 Ankang Lane, Dewai Avenue, Xicheng District, Beijing, 100088 China; 2 Advanced Innovation Center for Human Brain Protection, Capital Medical University, Beijing, 100069 China; 3 CAS Key Laboratory of Behavioral Science, Institute of Psychology & Magnetic Resonance Imaging Research Center, Institute of Psychology, Chinese Academy of Sciences, Beijing, 100101 China; 4 Department of Psychology, University of Chinese Academy of Sciences, Beijing, 100049 China

**Keywords:** Default mode network, Precuneus, Bipolar disorder, Resting-state functional magnetic resonance imaging, Spontaneous brain activity abnormalities

## Abstract

**Background:**

Theoretical and empirical evidence indicates the critical role of the default mode network (DMN) in the pathophysiology of the bipolar disorder (BD). This study aims to identify the specific brain regions of the DMN that is impaired in patients with BD.

**Methods:**

A total of 56 patients with BD and 71 healthy controls (HC) underwent resting-state functional magnetic resonance imaging. Three commonly used functional indices, i.e., fractional amplitude of low-frequency fluctuation (fALFF), regional homogeneity (ReHo), and degree centrality (DC), were utilized to identify the brain region showing abnormal spontaneous brain activity in patients with BD. Then, this region served as the seed region for resting-state functional connectivity (rsFC) analysis.

**Results:**

Compared to the HC group, the BD group showed reduced fALFF, ReHo, and DC values in the left precuneus. Moreover, patients exhibited decreased rsFCs within the left precuneus and between the left precuneus and the medial prefrontal cortex. Additionally, there was diminished negative connectivity between the left precuneus and the left putamen, extending to the left insula (putamen/insula). The abnormalities in DMN functional connectivity were confirmed through various analysis strategies.

**Conclusions:**

Our findings provide convergent evidence for the abnormalities in the DMN, particularly located in the left precuneus. Decreased functional connectivity within the DMN and the reduced anticorrelation between the DMN and the salience network are found in patients with BD. These findings suggest that the DMN is a key aspect for understanding the neural basis of BD, and the altered functional patterns of DMN may be a potential candidate biomarker for diagnosis of BD.

**Supplementary Information:**

The online version contains supplementary material available at 10.1186/s12888-024-05869-y.

## Background

Bipolar disorder (BD) is a lifelong mood disorder characterized by extreme fluctuations between mania and depression [1]. It affects more than 1% of the world’s population [2] and 0.6% of the Chinese population [3]. However, the pathophysiology of BD is poorly understood. Recent advances in the field of neuroimaging have enhanced our understanding on the neurophysiology of this disease [4]. In particular, the application of resting-state functional magnetic resonance imaging (rs-fMRI), abnormal local spontaneous brain activity, and resting-state functional connectivity (rsFC) have been repeatedly observed in patients with BD [5, 6].

These studies indicate that a brain network, called the default mode network (DMN), is particularly interested in the studies of BD. The DMN consists of the medial prefrontal cortex (MPFC), posterior cingulate cortex (PCC), precuneus [7], inferior temporal and superior frontal cortices, hippocampus, and parahippocampal cortices [8, 9]. When a person is awake, but not actively engaged in a goal-directed task, this network is active with a high degree of rsFC between regions [10]. The DMN is involved in multiple cognitive and affective functions, such as emotional processing, mind wandering, recollection of experiences, self-referential mental activity, social behavior, and episodic memory processes [7], all of which are affected to varying degrees in BD [11]. Broyd and colleagues [12] reviewed the putative mechanisms for DMN-related dysfunction in mental disorders, including BD, from two perspectives. On the one hand, altered rsFC within the DMN indicates that the integrity of DMN is impaired, which may be related to the deficit of working memory and attention, which is often observed in BD. On the other hand, in cognitive tasks that require attention, the activity of certain brain regions increases (referred to as task-positive networks), while the activity of the DMN decreases [13]. This indicates an antagonistic relationship between the two networks [14]. However, in mood disorders, this relationship may be compromised, potentially related to irregular transformation of introspective and extrospective thinking. The disbalance between the DMN and the salience network (SN) (one of the task positive networks) may be associated with emotion, cognitive, and psychomotor symptoms of BD [15].

Empirically, researches have consistently reported that different affective state in BD patients have rsFC abnormalities in the DMN [16–21]. For example, one study of drug naive or unmedicated patients with BD-II depression showed decreased rsFC of the PCC with the MPFC and precuneus [19]. Another study found that unmedicated patients with BD during depressive episode exhibited weaker rsFC in the left MPFC and right precuneus compared with healthy controls [22]. Additionally, decreased rsFC between the DMN and the SN was reported in patients with BD, although the sample included patients in different affective states (i.e., manic, depressive, mixed, and euthymic phases) [23]. In these previous studies, researchers often employed independent component analysis (ICA) to investigate the DMN [17, 20–22], or selected specific brain regions as seeds for the rsFC analysis of the DMN based on prior hypotheses [15, 19, 23, 24]. While these studies have yielded promising findings, no consistent evidence has emerged regarding the specific brain regions within the DMN that exhibit abnormal spontaneous activity in patients with BD.

In addition to the two methods, other analytical approaches can be applied to the studies of the DMN, such as fractional amplitude of low-frequency fluctuation (fALFF) [25], regional homogeneity (ReHo) [26], and degree centrality (DC) [27]. fALFF measures the magnitude of spontaneous fluctuations in the blood oxygen level-dependent signal (BOLD) at the voxel level, which is considered to reflect spontaneous neural activity [28]. ReHo is defined as the Kendall’s coefficient concordance (KCC) between the time series of voxels and their nearest neighbors, essentially a local FC that reflects the regulation and coordination of local neuronal activity [26]. DC assesses the summed FC of each individual voxel with all voxels in the brain [29], providing an opportunity for an unbiased general search of abnormalities in the entire connectivity matrix of the whole brain functional connectome [30]. With a whole-brain search strategy, these indices can identify abnormalities in the brain. Using these indices, researchers have already found decreased brain activity in the DMN of BD patients [31–33]. Therefore, the abnormal spontaneous brain activity in the DMN can be unbiasedly identified using these functional indicators. Finding the abnormal rsFC of the DMN in an unbiased approach is possible by using the regions identified by these functional indicators as the seed region in patients with BD. In a previous study, the researchers have found that the MPFC, an important node of the DMN, showed abnormal brain activity as indicated by ReHo and DC in patients with bipolar mania [34].

Our research hypothesis proposes that BD patients exhibit abnormalities in the DMN, resulting in atypical spontaneous brain activity and altered functional connectivity within distinct brain regions of the DMN. We employed a whole-brain search strategy to identify the regions with specific impairments within the DMN and to examine the rsFC of the DMN in the patients with BD. Specifically, from the point of view of local spontaneous brain activity and functional network, we used multiple brain functional indices including fALFF, ReHo, and DC to unravel abnormal brain spontaneous activities in BD. Subsequently, using the cluster that showed significant between-group differences as a seed, rsFC analyses, which reflect functional interactions of spatially distributed brain regions, were performed to further characterize the abnormal functional connectivity of the seed region in patients with BD.

## Methods

### Participants

A total of 150 participants were recruited from the community (85 HCs) and the Beijing Anding Hospital (65 patients with BD); the sample is the same as in our previous publication [35]. In summary, the patients and HC were matched for age, gender, and education. Twenty-three subjects (nine patients with BD and 14 HCs) were excluded from the final analysis due to excessive head motion (details are shown in the “fMRI data preprocessing”). All patients satisfied the diagnostic criteria of the Structured Clinical Interview for DSM-IV (SCID) [36], but in this study, we did not differentiate between BD-I and BD-II. Our inclusion and exclusion criteria were described in our previous study [35]. In this experiment, the Young Mania Rating Scale (YMRS) [37] scores of all patients were less than 7, thereby indicating that all patients were not in a manic mood state. And the Hamilton Depression Rating Scale (HAMD) [38] was used to assess patients’ depressive symptoms. Patients with HAMD scores of ≤ 7 were classified as euthymic patients (EP, *n* = 30), while those patients with scores above this threshold were considered to be in a depressive state (DP, *n* = 26).

We focused on patients with BD in a depressive mood state or a euthymic mood state because BD patients spend more than 80% of their time in a non-manic mood state. The current study has been approved by the Institutional Review Board of Brain Image Center, Beijing Normal University and Beijing Anding Hospital, Capital Medical University. Written informed consents were obtained from all subjects prior to participation in this study.

### fMRI data acquisition

All imaging data were acquired at the Brain Imaging Center of the Beijing Normal University by using a Siemens TIM Trio 3T scanner (Siemens, Erlangen, Germany). Resting state functional images (240 volumes) were acquired initially, followed by the T1 images. During scanning, subjects were asked to try not to swallow or move their body, especially their heads. During resting state, subjects were also instructed to close their eyes, relax, and remain awake. Resting state functional images were collected using echo-planar imaging (EPI) sequence, as follows: axial scanning, repetition time (TR) = 2000 ms, echo time (TE) = 30 ms, flip angle = 90°, field of view (FOV) = 200 × 200 mm^2^, matrix size = 64 × 64, slices = 33, interlaced scanning, slice thickness = 3.5 mm, and gap = 0.7 mm. T1 images were collected using T1-weighted sagittal 3D magnetization-prepared rapid gradient echo (MPRAGE) sequence, as follows: TR = 2530 ms, TE = 3.39 ms, flip angle = 7°, FOV = 256 × 256 mm^2^, matrix size = 192 × 256, slices = 128, thickness = 1.33 mm, and voxel size = 1.33 × 1 × 1 mm^3^.

### fMRI data preprocessing

Resting state fMRI date was preprocessed in the toolbox for the Data Processing and Analysis for (Resting State) Brain Imaging (DPABI v3.1) [39]. The preprocessing steps included removing the first five time points, slice timing, realignment, detrending, removing the head motion effect (using Friston 24-parameter model) [40], regressing out nuisance covariates (the white matter signals, the cerebral spinal fluid signals, and the global mean signals), normalizing the MNI space (using T1 images), resampling (voxel size = 2 × 2 × 2 mm^3^), smoothing (FWHM = 4 mm, only for the fALFF and rsFC analysis), and band-pass filtering (0.01–0.1 Hz, only for the ReHo, DC, and rsFC analysis). In addition, we used volume-based framewise displacement (FD) [41] to quantify head motion. FD reflects head motion from one volume to the next, and a volume with FD < 0.2 indicates a good time point. Mean FD is calculated by the average of the sum of the absolute values of the differentiated realignment estimates (by backwards differences) at every timepoint [42]. Subjects would be excluded if their mean FD exceeded three standard deviations beyond the mean value of the entire sample or the number of good time points was less than 120.

### fMRI data analysis

First, three indices of the fALFF, the ReHo, and the DC were used to unravel abnormal brain activities in the patients with BD. Subsequently, using the overlapped cluster of the significant brain region found in the analysis as seed, rsFC analysis was performed to explore possible brain regions, which showed abnormal rsFC with the overlapped cluster in patients with BD. Finally, two additional strategies were used to reidentify the seed region and then confirmed our rsFC analysis.

#### fALFF, ReHo, and DC calculation

Using the preprocessed resting state fMRI data, we calculated the fALFF, ReHo, and DC values for each subject in DPABI v3.1. All values were calculated at voxel level. The fALFF value was calculated using the signal strength of the low frequency range (i.e., 0.01–0.1 Hz) to divide the detectable entire frequency range [25]. The ReHo value, also called the Kendall’s coefficient of concordance (KCC), of the time series of a given voxel with its nearest neighbors (27 voxels were considered) was calculated to generate individual ReHo maps [26]. The DC value of a certain voxel was the sum of the Pearson’s correlation coefficients of all possible pairs of voxels (correlation threshold of r_0_ was set at 0.2) [27, 43]. Lastly, the fALFF, ReHo, and DC maps for each subject were converted into *Z*-score maps, respectively. For the ReHo and DC maps, smoothing (FWHM = 4 mm) was performed.

#### Functional connectivity analysis

This analysis was performed in DPABI v3.1. The overlapped brain region, which showed significant group differences in fALFF, ReHo, and DC values between patients with BD and HC, was used as a seed in the following functional connectivity analysis. First, time series of each voxel was extracted within the seed. Second, the mean time series of the seed was calculated by averaging each voxel’s time series. Third, the Pearson correlation coefficients of the mean time series of the seed and all other voxels’ time series were calculated. They were used to construct each subject’s rsFC map. Finally, each subject’s rsFC map was converted into Z-score map.

## Statistical analyses

For categorical variables of demographic and clinical data, we used chi-square test for group comparison. For continuous variables, we first used the Shapiro-Wilk test for normality testing. If the variable followed a normal distribution, we used a two sample T-test for group comparison. If it did not follow a normal distribution, the Man-Whitney U test was used to compared the differences between the patients with BD and the HC. The above processes are all calculated in SPSS 20.0.

For each functional index (i.e., the fALFF, the ReHo, the DC, and the rsFC), analyses of the differences between the two groups were conducted in SPM12 (Wellcome Department of Cognitive Neurology, London, UK). In these analyses, two-sample T tests were used with age, gender, education, and mean FD controlled. The significance level was set at the combination of voxel-level uncorrected *P* < 0.001 and cluster-level Family Wise Error (FWE)-corrected *P* < 0.05. And in order to further investigate the correlation between imaging indicators and clinical symptoms, we used Pearson correlation analysis to study the correlation between the fALFF, DC, and ReHo of the clusters showing significant inter group differences and the total HAMD score. The significance level was set at 0.05/3 = 0.017. These statistical analyses were limited in a pre-made gray mask. The gray matter mask was generated by averaging the gray matter masks segmented from the T1 images of each subject.

## Confirmation analyses of the rsFC

To validate the findings obtained in the rsFC analysis, we used two strategies to confirm the findings. (i) We used the significant cluster obtained in the group analyses of the fALFF and the DC, but not the ReHo (due to the negative finding in a strict statistical threshold) to calculate the overlapped cluster. The overlapped cluster was then used as a seed in the rsFC analysis. (ii). We attempted to map our interested brain regions identified in the analysis of the fALFF, ReHo, and DC to the Brainnetome atlas [44]. Then, we selected the corresponding brain region of the atlas as seeds for further rsFC analysis. This method reduces the subjective bias in selecting the seed region and makes our findings more likely to be replicated by future studies.

In order to find possible state-specific alterations of different affective status in BD, we compared the whole brain functional connectivity maps computed with the precuneus as the seed between the EP and HC and between the DP and HC, and further seek to correlate the significant clusters obtained with the clinical features, respectively. Detailed methods could be found in the supplementary materials.

## Results

### Demographic and clinical characteristics

Table 1 summarizes the demographic and the clinical data of all subjects. We found that age, HAMD, and mean FD did not follow a normal distribution (all *P* < 0.05). Therefore, median and upper and lower quartiles were used for representation. The two groups were comparable in terms of age, gender, education, and mean FD (all *P*-values > 0.05). The HAMD scores of the BD group were significantly higher than those of the HC group (Z = -7.12, *P* < 0.001). As for drug use, the majority of patients was treated with at least one type of psychotropic medication (Table 1).

**Table 1:** Demographic and clinical feature of participants

	Bipolar disorder (*N* = 56)	Healthy controls (*N* = 71)	Statistics	*P*
**Age**	27(22,33.75)	26(23,35)	-0.37 (Z)	0.713
**Sex (male/female)**	35/21	34/37	2.69 (χ^2^)	0.101
**Education** ^**a**^	7/22/12/15	6/15/18/32	7.08 (χ^2^)	0.069
**Duration of illness (m)**	45.78 ± 68.49	NA	NA	NA
**HAMD**	5(2,13)	0(0,1)	-7.12 (Z)	< 0.001*
**Mean FD**	0.13(0.11,0.17)	0.13(0.10,0.16)	-1.23 (Z)	0.219
**Medication**				
**Unmedicated** **Mood stabilizers** **Antipsychotics** **Antidepressants**	5 28 27 19	NA NA NA NA	NA NA NA NA	NA NA NA NA

NA: not applicable

HAMD: Hamilton Depression Scale; FD: Framewise displacement

^a^ The education level of all subjects was divided into the following categories: junior high school / senior high school or special secondary school / junior college / Bachelor degree or above

*P < 0.05

### fALFF, ReHo and DC analyses

**Fig. 1:**
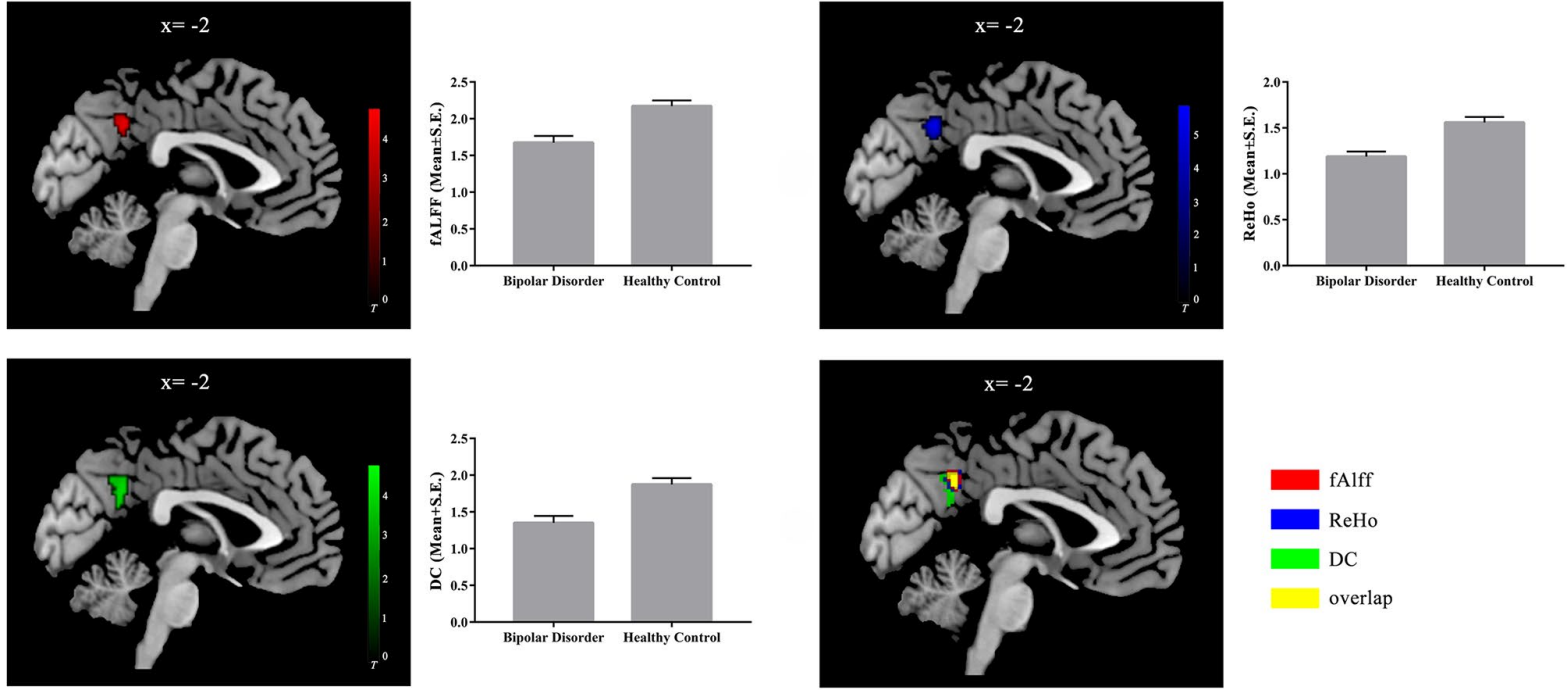
fAlff, ReHo, and DC maps between-group differences in each functional index. The patients with BD showed decreased fAlff (in red), ReHo (in blue), and DC (in green) in the left precuneus. The overlapped cluster (47 voxels) across these functional indices is represented in yellow

The fALFF, ReHo, and DC map within each group are shown in Figure S1. The whole-brain analysis found that the BD group showed significantly lower fALFF and DC in the left precuneus, which was the only result in both tests. As for the ReHo, though the peak voxel reached the significant level of voxel-level FWE-corrected *P* < 0.05, the largest cluster, where the peak voxel was located did not reach our significant level. Thus, we used a relatively less stringent criteria for our ReHo data analysis, i.e., we regarded the largest cluster as an interesting cluster (*P* < 0.001, cluster-level uncorrected). Then, the significant clusters were overlapped, and the common overlapped cluster consisted of 47 voxels, located in the left precuneus (Fig. 1 and Table 2). Furthermore, we did not find significant statistical correlation between the fALFF, DC, and ReHo of the precuneus and total HAMD scores (all *P* > 0.017) in BD group.

**Table 2:** Between-group differences in the fALFF, ReHo and DC analyses between the patients with BD and the healthy controls

		Brain region	Hemisphere	BA	MNI coordinates	Peak T values		Cluster size	Cluster-level *P*_FWE_
**fALFF**	Patients < Controls	Precuneus	Left	31/7	0, -54, 36	4.49		69	0.004
**ReHo** ^**a**^	Patients < Controls	Precuneus	Left	31/7	0, -52, 36	5.29		106	0.135
**DC**	Patients < Controls	Precuneus	Left	31/7	-10, -58, 36		4.50	187	0.017

^a^ voxel-level threshold was *P* < 0.001, cluster-level uncorrected

BD: bipolar disorder; fALFF: fractional amplitude of low-frequency fluctuation; ReHo: regional homogeneity; DC: degree centrality; BA: Brodmann area; MNI: Montreal Neurological Institute

### Seed-based rsFC analysis

**Fig. 2:**
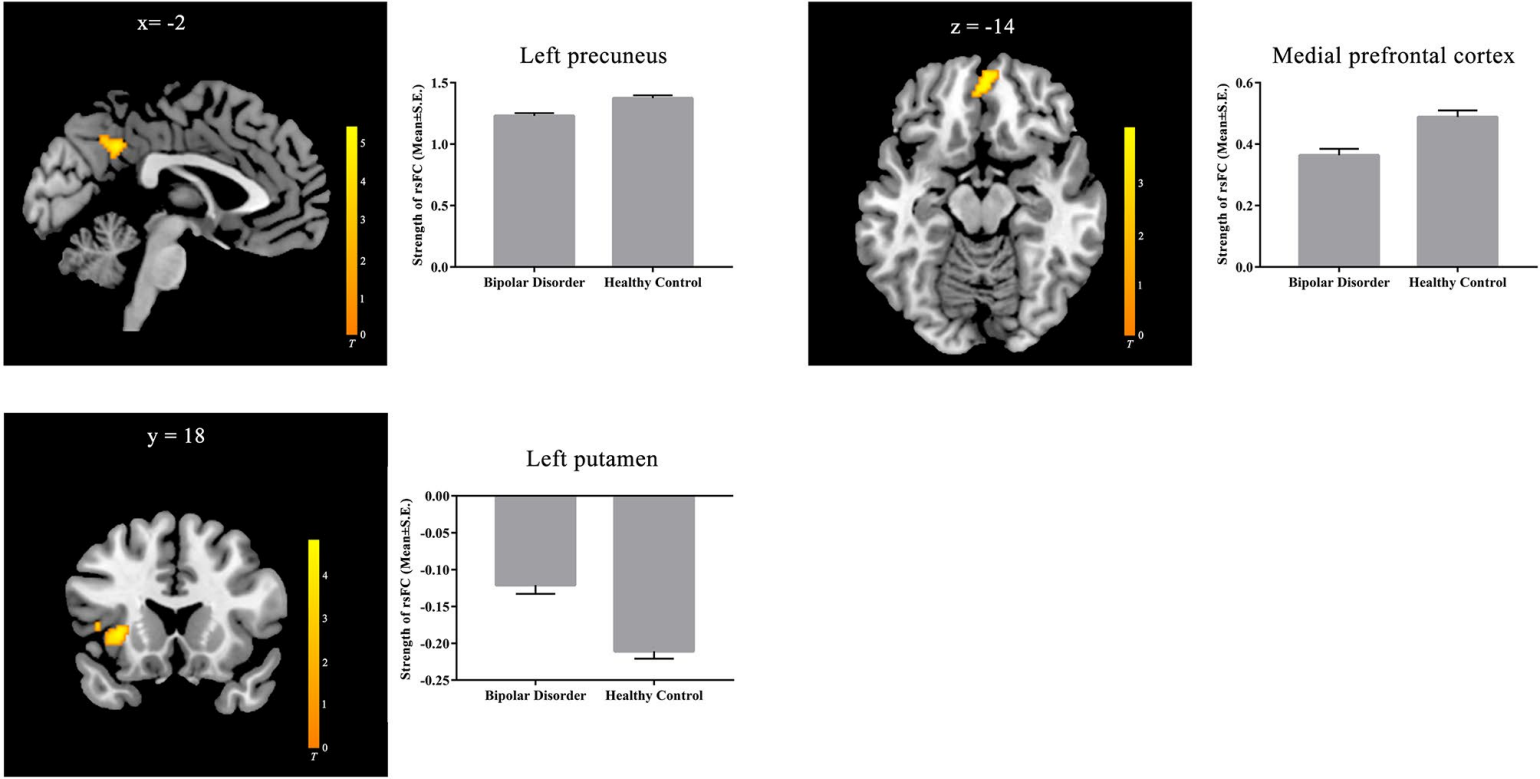
rsFC (using the overlapped 47 voxels as a seed) map between-group differences in the rsFC map. The left precuneus and the medial prefrontal cortex whose rsFC with the left precuneus were decreased, and the left putamen whose rsFC with the left precuneus was increased in patients with BD

The rsFC map within each group is also shown in Figure S2. Using the overlapped 47 voxels found above as the seed, we found that the BD group showed decreased rsFC of the seed with the left precuneus and the MPFC and increased rsFC (i.e., the decreased negative rsFC) of the seed with the left putamen (extended to the left insula) (Fig. 2and Table 3).

**Table 3:** Between-group differences in the rsFC analyses between the patients with BD and the healthy controls

Seeds				Brain regions	Hemisphere	BA	MNI coordinates	Peak T values	Cluster size	Cluster-level *P*_FWE_
**47 voxels in the precuneus overlapped across fALFF, ReHo and DC**		Patients > Controls	Putamen/insula		Left	47/13	-32, 18, 0	4.92	217	<0.001
		Patients < Controls	Precuneus		Left/Right	31/7	-2, -50, 36	5.29	97	0.014
			Medial prefrontal cortex		Left/Right	11	2, 52, -14	3.97	89	0.023
**57 voxels in the precuneus overlapped between fALFF and DC**		Patients > Controls	Putamen/insula		Left	47/13	-32, 18, 0	4.96	197	< 0.001
		Patients < Controls	Precuneus		Left/Right	31/7	-2, -50, 36	5.50	109	0.006
			Medial prefrontal cortex		Left/Right	11	2, 52, -14	4.03	89	0.023
**Precuneus in the Brainnetome region** **(No. 153 + 154)**		Patients < Controls	Medial prefrontal cortex		Left/Right	11	2, 52, -14	4.50	126	0.014

BD: bipolar disorder; fALFF: fractional amplitude of low-frequency fluctuation; ReHo: regional homogeneity; DC: degree centrality; BA: Brodmann area; MNI: Montreal Neurological Institute

### Confirmation analyses of the rsFC

For the first confirmation analysis, when we used the significant region found in the fALFF and the DC analysis to identify the overlapped region, we found a cluster with 57 voxels located in the left precuneus (Figure S3). Using this cluster as a seed, the rsFC map within each group is shown in Figure S4. We found significantly decreased rsFC of the seed with the left precuneus and the MPFC and increased rsFC (i.e., the decreased negative rsFC) of the seed with the left putamen (Figure S5 and Table 3).

For the second confirmation analysis, we found that the significant clusters of every single functional index (i.e., fALFF, ReHo, and DC) and the overlapping clusters corresponded consistently to the brain regions No. 153 and 154, also named bilateral area 31 (A31) of precuneus, in the Human Brainnetome atlas (Figure S6-A). Then, we used the sum of the brain region Nos. 153 and 154 as a seed in the following rsFC analysis. The rsFC map within each group is shown in Figure S6-B. The whole-brain analysis found a significantly decreased rsFC of the seed with the MPFC (Figure S6-C and Table 3).

Furthermore, in the subgroup analysis, we observed that individuals of the EP group exhibited increased rsFC (indicating decreased negative rsFC) between the precuneus and the left putamen (extended to the left insula), in comparison to HC (Figure S7, Table S1). Subsequently, upon reducing the threshold, we identified that the outcomes for both subgroups were consistent with the comparisons between individuals with BD and HC. Detailed results were presented in the supplementary materials.

## Discussion

We used three main functional indices (fALFF, ReHo, and DC) to measure the spontaneous activity from the local activity, local connectivity to network-level functional connectivity. Consistent with our formulated research hypothesis, our findings corroborate the presence of diminished spontaneous activity within the left precuneus, a pivotal node within the DMN, among patients diagnosed with BD. Furthermore, using this overlapped region in the left precuneus as a seed for rsFC, we found the decreased rsFC within the DMN, indicated by the decreased rsFC with the left precuneus and decreased rsFC between the seed precuneus and the MPFC in patients with BD. In addition, we found decreased negative functional connectivity between the left precuneus and the core regions in the SN (i.e., the left putamen/insula), suggesting decreased anticorrelation between the DMN and the SN in patients with BD.

### Altered spontaneous activity of the DMN

We used multiple functional indices and found that the patients with BD exhibited decreased fALFF, ReHo, and DC values in the left precuneus (a specific brain region of the DMN) compared with healthy controls. The decrease in the fALFF indicates reduced local BOLD signal fluctuations of the precuneus [25, 45]. The decrease in the ReHo indicates decreased synchronizing ability in relevant voxels within the precuneus [26], and the decrease in DC indicates reduced functional connections of the precuneus with other voxels in the brain [46]. The co-occurrence of abnormalities in fALFF and DC within the precuneus signifies disrupted spontaneous brain activity in this specific brain region. This study revealed a significant correlation (*r* = 0.671, *P* < 0.001) between fALFF and DC values in the precuneus across all samples, consistent with findings from Yan’s research that also reported a strong correlation between these two metrics [47]. Our findings are consistent with most previous studies [32]^,^ [48]^,^ [49], which found decreased spontaneous brain activities of the left precuneus in patients with BD. However, another study, which only recruited 17 bipolar depression patients and 16 HCs, reported increased ReHo values of the left precuneus in patients [50]. Furthermore, in this study, there is no correlation between the local activity of precuneus and clinical symptoms, suggesting that clinical symptoms may not affect the local activity of precuneus.

The voxel-based hemodynamic characteristics of BOLD signals at rest are highly likely to reflect metabolic needs, and some researchers have even suggested that fALFF and ReHo could be considered biomarkers of metabolism [51]. For example, a study found a spatial correlation between PET and rs-fMRI indicators (i.e., fALFF, ReHo, DC), especially in the DMN where the correlation is strongest [52]. Therefore, the dysfunction detected by these functional indices may be explained by the abnormal metabolism in this region. One PET study found reduced metabolism of the left precuneus in the old euthymic patients with BD [53]. The precuneus is located in the posterior medial part of the parietal lobe, which is extensively connected with cortex and subcortical brain areas [54]. The precuneus is associated with emotion regulation [55], and is especially sensitive to negative emotion. A left lateralized activation of the precuneus was found when participants viewed negative images [56] or when they were asked to increase their negative emotions when facing aversive stimuli [57]. One study using a negative emotion task found reduced activation of the left precuneus during negative images condition in the euthymic BD-I patients, with respect to controls [58]. This finding suggested that decreased brain activity of the left precuneus in patients with BD may somehow interfere with their ability to process emotional contents. The results found in the current resting-state fMRI study highlighted the importance of the precuneus, especially its left part, in the pathogenesis of BD. Thus, the patients’ left precuneus was unable to maintain normal signal fluctuation, synchronizing ability, and connectivity with other brain regions.

### Decreased rsFC within the DMN

The followed rsFC analysis found hypoconnectivity within the left precuneus in the patients with BD. The results were consistent with previous studies [59, 60]. Wang et al. [44] used FC strength (FCS) to measure the whole-brain FCS patterns, and found that unmedicated patients with BD-II depression showed decreased FCS in the DMN (including the left precuneus). Khadka et al. [60] using ICA found that the rsFC in the posterior DMN consisting mainly of bilateral precuneus was significantly decreased in patients with psychotic BD-I than the HC. Combining these findings with our results, we speculated that the decreased local spontaneous activity of the left precuneus may account for its decreased rsFC. In addition, the precuneus is a functional core of the DMN [61]. It not only shows the highest resting metabolic rate within the network [62], but has widespread connectivity with other brain regions [54]. It suggested that the anomaly of precuneus could have an important effect on the overall functional abnormality of the DMN [54, 61]. The patients with BD also exhibited reduced rsFC between the left precuneus and the MPFC, which is another core node of the DMN [63]. This result was repeated in our confirmation analyses. The current result was, to some extent, consistent with previous report of Wang et al. [64], although two additional studies using smaller sample size (the number of patients with BD is 15 and 30) did not find positive results [24, 65]. In Wang’s study, unmedicated patients with BD under depressive episode were included, and patients showed disrupted intramodular connectivity within the DMN. The reduced rsFC between the MPFC and the left precuneus reflected weaker synchronization of spontaneous neural activities within the DMN, which could reduce the efficiency of the information communication and integration of the network [66]. The precuneus is involved in self-centered mental imagery strategies and episodic memory retrieval [54]. The MPFC is one of the most clearly delineated regions in terms of its functional roles; it serves an important role in emotional experience, social cognition, memory, and decision making [67, 68]. Some researchers suggested that the abnormal rsFC between the anterior (e.g., the MPFC and the superior frontal gyrus) and the posterior (e.g., the PCC/precuneus) components of the DMN sever as the neural basis for the defects of attention and working memory [12]. The patients with BD have been reported to have these cognitive abnormalities [69]. One study on the relationship between the DMN and clinical symptoms of BD found that the decreased rsFC within the posterior DMN in patients with BD depression might be associated with rumination [19], which is a typical symptom for depressed patients. Simply put, rumination is the process in which a ruminant animal continuously regurgitates partially digested food from its stomach for further chewing. From a psychopathological perspective, rumination is defined as a distress response pattern characterized by passive and persistent contemplation of one’s distressing symptoms, their origins, and repercussions, without engaging in active problem-solving to address the underlying causes of the distress [70]. When we engage in rumination, the brain activity of DMN increases [71]. From this aspect, it would be reasonable to speculate that the decreased rsFC between the MPFC and the precuneus may result in the cognitive dysfunction and clinical symptoms for patients with BD. In summary, our findings with all previous studies support that the functional dysconnectivity within the DMN may play a relatively important role in the neural basis of BD.

### Decreased anticorrelation between DMN and SN

Decreased negative connectivity was found between the left precuneus and the left putamen/insula, the main nodes of the SN [3, 72], in the patients with BD. This finding reflects the decreased anticorrelation between the DMN and the SN.

In healthy population, resting-state fMRI signals of the DMN are anticorrelated with that of task-positive networks, such as the SN [73–75]. The SN generates control signals and causally mediates the “switching” between the DMN and the central executive network [76]. This condition leads to a switch between their functions of introspective and extroversive attention orientation, and then allows individuals to stay vigilant when meeting unexpected environmental events [12]. This anticorrelation between DMN and SN has even been proven more important than the DMN’s own activity [77]. The decreased anticorrelation between these networks may result in the reduction of introspective thinking and attention deficit, which could be the potential interference source of goal-directed behavior [12]. Weaker anticorrelation between the DMN and the SN was also found in many other mental disorders [73, 78]. Our result expanded previous findings of disruption of the DMN-SN functional connectivity in mental disorders [23] by showing that the negative rsFC between the left precuneus and the left putamen/insula was decreased in patients with BD.

Moreover, it is noteworthy that findings obtained from the subgroup analysis were aligned with those obtained from the comparisons between individuals with BD and HC, especially increased rsFC between the precuneus and the left putamen (extended to the left insula). It is suggested that the increased precuneus-left putamen connection may be not unaffected by patients’ mood states, and may be a trait-like feature for BD.

### Limitations

Some limitations are as follows. Firstly, although the patients were not in a manic mood state, our sample was still heterogeneous in the subtypes or the different affective status of the illness. Future work should recruit patients in the same mood state (mania, depression, or euthymia) to identify whether the significant results were state-dependent or trait-like characters of BD. Secondly, most patients had taken different types or dosages of drugs. Although no consistent effect of any particular drug was found on BOLD signals, different drugs or dosages may influence BOLD signals in different ways [79], potentially confounding our results. In future studies, unmedicated patients with BD should be recruited or the types and dosages of medication use should be strictly controlled. Thirdly, our study did not examine whether these brain changes could account for any clinical or cognitive symptoms, such as rumination, or whether these brain abnormalities changed over time. Further studies are needed to address these issues.

## Conclusions

In summary, using multiple functional indices, we found decreased spontaneous brain activity in DMN, particularly in the left precuneus. We also observed decreased functional connectivity within the DMN and reduced anticorrelation between the DMN and the SN in patients with BD. These findings suggest that the DMN is a key aspect for understanding the neural basis of BD. The altered functional patterns of the DMN may be a potential candidate biomarker for the diagnosis of BD.

### Electronic supplementary material

Below is the link to the electronic supplementary material.

Supplementary Material 1

## Data Availability

Data are available from the first and the corresponding authors.

## Abbreviations

DMN: the default mode network
SN: salience network
BD: bipolar disorder
HC: healthy controls
EP: euthymic patients with bipolar disorder
DP: depressed patients with bipolar disorder
rs-fMRI: resting-state functional magnetic resonance imaging
fALFF: ractional amplitude of low-frequency fluctuation
ReHo: regional homogeneity.
DC: degree centrality
rsFC: resting-state functional connectivity
MPFC: medial prefrontal cortex
PCC: posterior cingulate cortex
YMRS: Young Mania Rating Scale
HAMD: Hamilton Depression Rating Scale
EPI: echo-planar imaging
TR: repetition time
TE: echo time
FOV: field of view
MPRAGE: magnetization-prepared rapid gradient echo
KCC: Kendall’s coefficient of concordance
FWE: Family Wise Error
FCS: FC strength
