# Supplementary material for: Disrupted default mode network connectivity in bipolar disorder: a resting-state fMRI study
Source: BMC Psychiatry. 2024 Jun 7;24:428. doi: 10.1186/s12888-024-05869-y (PMC11157927; doi:10.1186/s12888-024-05869-y)
Supplement: Supplementary file 1 — Supplementary Material 1 [file 12888_2024_5869_MOESM1_ESM.pdf]

Supplementary materials

Results about the exploratory analysis of the subgroups in BD

We used the overlapped cluster (i.e., the precuneus) obtained from the between-group analyses of the fALFF, ReHo, and DC as the seed, and calculated its functional connectivity with the whole brain. Then, we compared functional connectivity maps between EP and HC and between DP and HC. Our significant level was set at voxel-level uncorrected $P<0.001$ and cluster-level FWE corrected $P<0.025$ (Because we compared functional connectivity maps twice, so the significant level of the cluster level should be 0.05/2=0.025).

The results showed that compared with HC, EP showed increased functional connectivity (i.e., negative functional connectivity decreased) between the Precuneus and the left putamen (extended to the left insula) (**FigureS7, TableS1**). The significant cluster was close to the cluster found in the comparison between the whole patients with BD and HC. Subsequently, we used the Pearson correlation analysis to calculate the correlation between functional connectivity found above and the HAMD scores in both the BD and HC groups respectively, however, we did not find any statistically significant correlation (All $P > 0.05$).

No significant cluster was found showing decreased functional connectivity in the EP group when comparing with the HC group. However, we noticed that, when we lower the significant threshold (i.e., only requiring voxel-level threshold of $P_{\text{uncorrected}} < 0.005$) and restricted our results within the significant cluster found in the comparison of the whole patients with BD and HC, we could indeed observe that some clusters exhibiting decreased functional connectivity in the EP group. For the Precuneus, there were two clusters (42 voxels and 2 voxels, respectively), for the MPFC, there were also two clusters (10 voxels and 13 voxels, respectively), though they all did not survived the cluster-level multiple comparison correction.

For the comparation of DP and HC, no significant between-group difference was found. However, again, when we lower the significant threshold (i.e., only requiring voxel-level threshold of $P_{\text{uncorrected}} < 0.005$) and restricted our results within the significant cluster found in the comparison of the whole patients with BD and HC, we could indeed observe that some clusters left. For the DP-HC, four clusters were left in the putamen (48 voxels, 5 voxels, 1 voxel and 2 voxels); for the HC-DP, one cluster was left in the Precuneus (73 voxels) and two cluster were left in the MPFC (58 voxels and 2 voxels, respectively), though they all did not survived the cluster-level multiple comparison correction.

Figure S1. fAlff, ReHo, and DC maps within each group

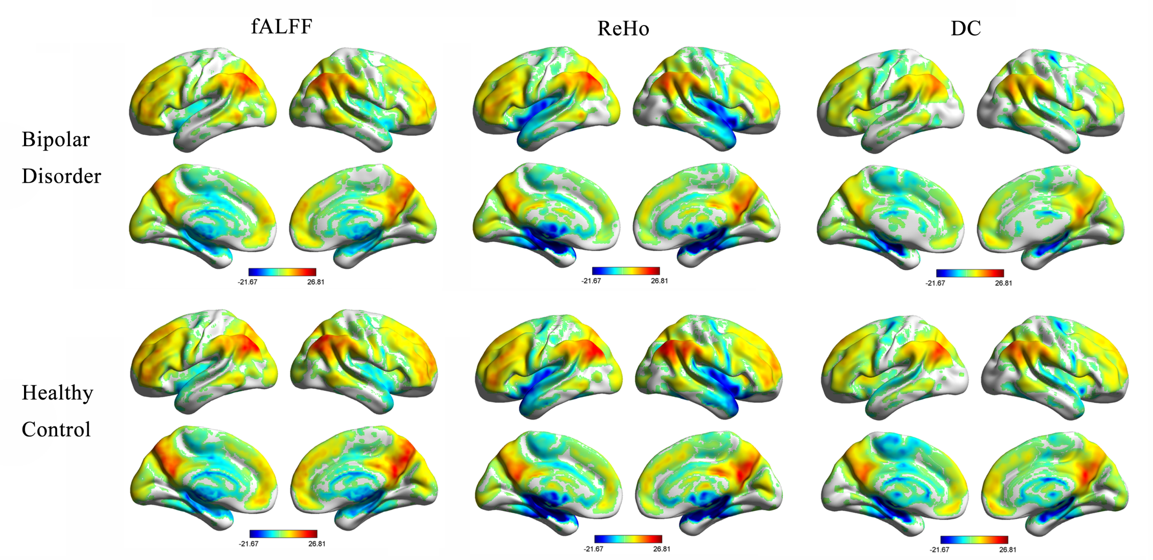

Figure S2. rsFC (using the overlapped 47 voxels as a seed) map within each group

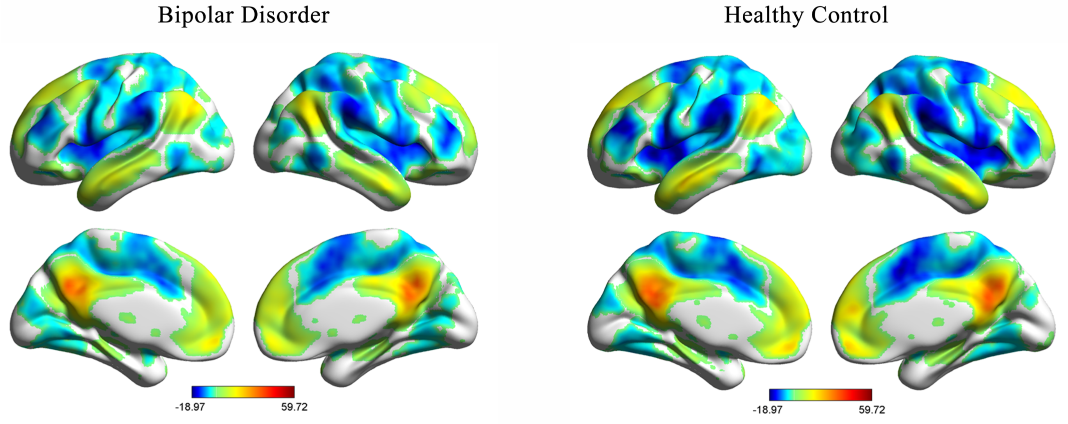

Figure S3. Overlapped cluster (57 voxels) between the fALFF and DC analyses. The patients with BD showed decreased fAlff (in red) and DC (in green) in the left precuneus. The overlapped cluster (57 voxels) was represented in yellow.

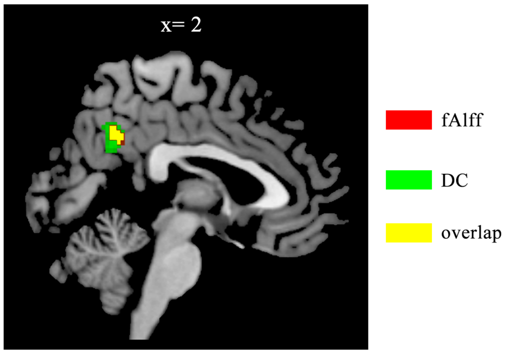

Figure S4. rsFC (using the overlapped 57 voxels as a seed) map within each group

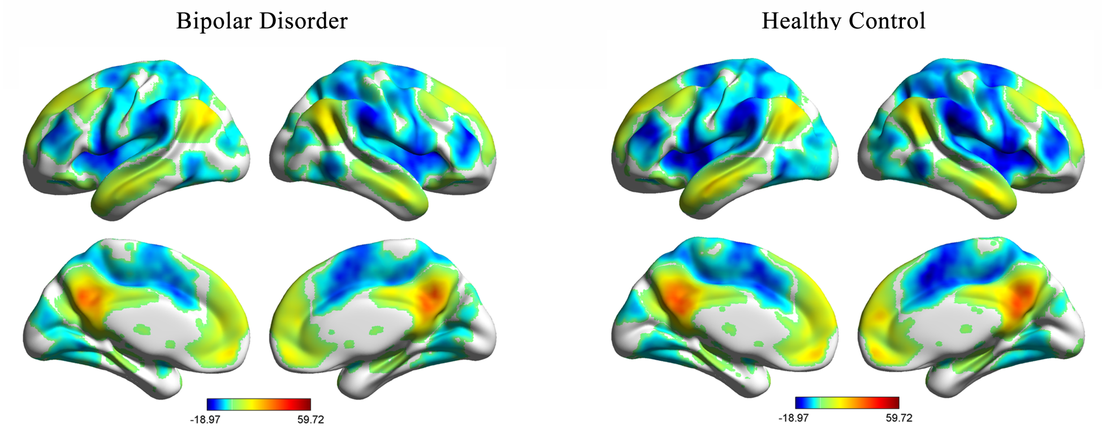

Figure S5. rsFC (using the overlapped 57 voxels as a seed) map between-group differences in the rsFC map. The left precuneus and the medial prefrontal cortex whose rsFC with the left precuneus were decreased, and the left putamen whose rsFC with the left precuneus was increased in patients with BD.

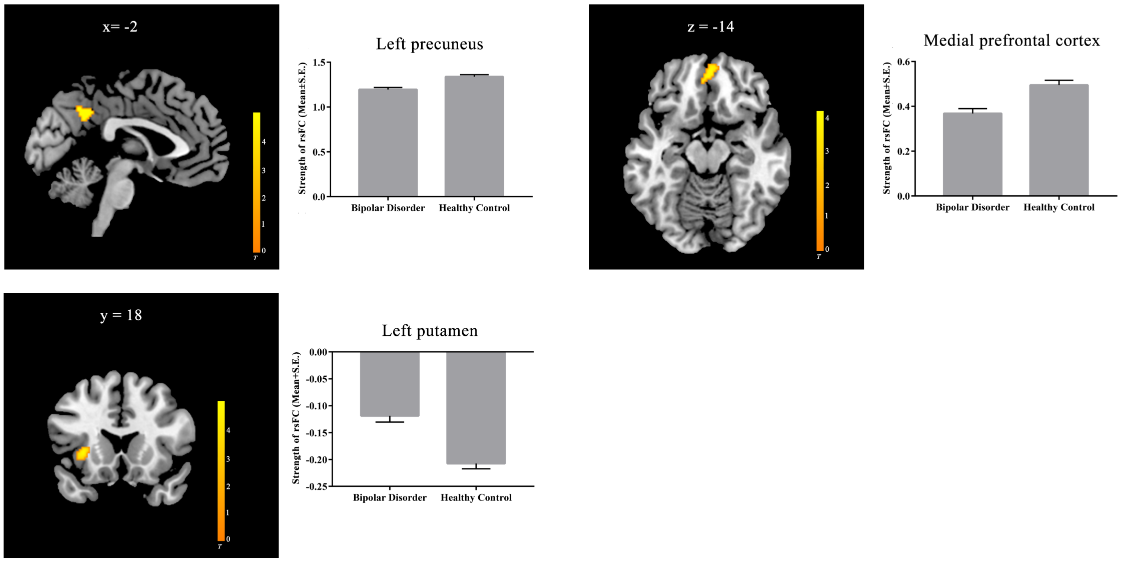
x= -2
Left precuneus
Strength of rsFC (Mean±S.E.)
Bipolar Disorder
Healthy Control
z = -14
Medial prefrontal cortex
Strength of rsFC (Mean±S.E.)
Bipolar Disorder
Healthy Control
y = 18
Left putamen
Strength of rsFC (Mean±S.E.)
Bipolar Disorder
Healthy Control

Figure S6. Between-group difference in the rsFC of the left precuneus (No. 153 and 154 in the Brainnetome atlas). Panel A showed the overlapped region between the combined brain regions No. 153 and 154 in the Brainnetome atlas (in red) and the cluster identified by each of functional index (in yellow). Panel B showed the rsFC map within each group. Panel C showed the region whose rsFC with the left precuneus was decreased in patients with BD.

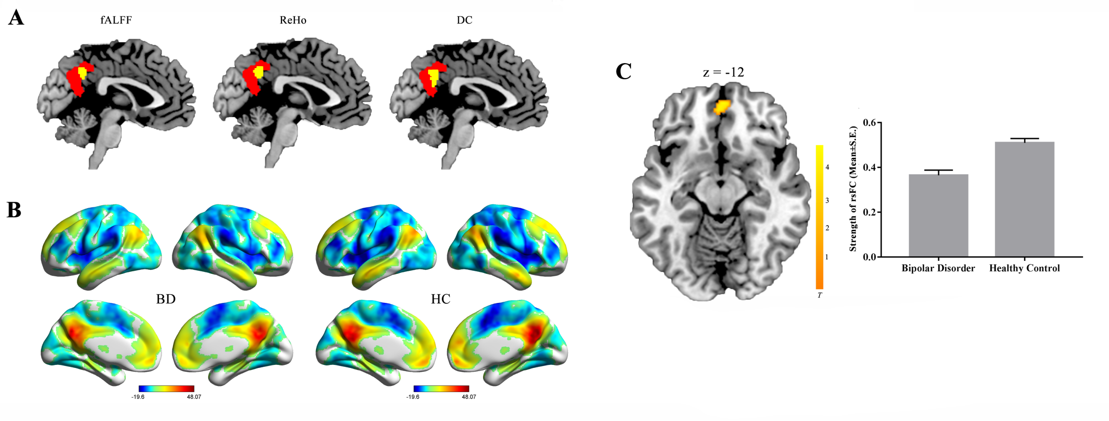

Figure S7. rsFC (using the overlapped cluster obtained from the between-group analyses of the fALFF, ReHo, and DC as the seed) map between-group differences in the rsFC map. Compared with HC, EP showed increased rsFC (i.e. negative functional connectivity decreased) between Precuneus and the left putamen (extended to the left insula)

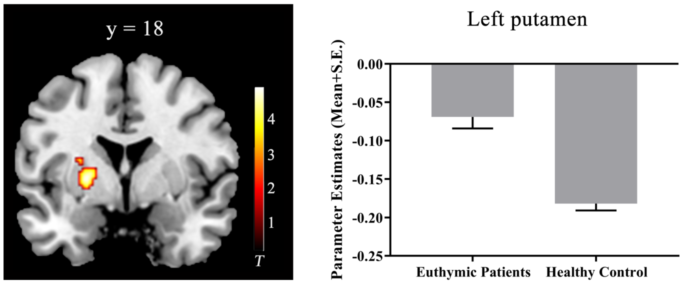

Table S1. Between-group differences in the rsFC analyses between the patients with euthymic BD and the healthy controls

**Seeds**		**Brain regions**	**Hemisphere**	**BA**	**MNI coordinates**	**Peak T values**	**Cluster size**	**Cluster-level P_{FWE}**
**47 voxels in the precuneus overlapped across fALFF, ReHo and DC**	Patients> Controls	Putamen/ insula	Left	47/13	-32, 16, 0	4.81	160	<0.001

BD: bipolar disorder; fALFF: fractional amplitude of low-frequency fluctuation; ReHo: regional homogeneity; DC: degree centrality; BA: Brodmann area; MNI: Montreal Neurological Institute.
